# Impact of Statewide Mandatory Medicaid Managed Care (SMMC) Programs on Hospital Obstetric Outcomes

**DOI:** 10.3390/healthcare10050874

**Published:** 2022-05-09

**Authors:** Hasan Symum, José Zayas-Castro

**Affiliations:** Department of Industrial and Management Systems Engineering, University of South Florida, Tampa, FL 33620, USA; josezaya@usf.edu

**Keywords:** cesarean, preterm birth, readmission, VBAC, managed care

## Abstract

The state of Florida implemented mandatory managed care for Medicaid enrollees via the Statewide Medicaid Managed Care (SMMC) program in April of 2014. The objective of this study was to examine the impact of the implementation of the SMMC program on the access to care and quality of maternal care for Medicaid enrollees, as measured by several hospital obstetric outcomes. The primary data source for this retrospective observational study was the Hospital Cost and Utilization Project (HCUP) all-payer State ED (SED) visit and State Inpatient Databases (SIDs) from 2010 to 2017. The primary health outcomes for obstetric care were primary cesarean, preterm birth, postpartum preventable ED visits, postpartum preventable readmissions, and vaginal delivery after cesarean (VBAC) rates. Using difference-in-differences (DID) estimation, selected health outcomes were examined for Florida residents with Medicaid beneficiaries (treatment) and the commercially insured population (comparison), before and after the implementation of SMMC. Improvement in disparities for racial/ethnic minority Medicaid enrollees was estimated relative to whites, compared to the relative change among commercially insured patients. From the DID estimation, the findings showed that SMMC is statistically significantly associated with a higher reduction in primary cesarean rates, preterm births, preventable postpartum ED visits, and readmissions among Medicaid beneficiaries relative to their commercially insured counterparts. However, this study did not find any significant reduction in racial/ethnic disparities in obstetric outcomes. In general, this study highlights the impact of SMMC implementation on obstetric outcomes in Florida and provides important insights and potential scope for improvement in obstetric care quality and associated racial/ethnic disparities.

## 1. Introduction

Medicaid in the United States plays a critical role in ensuring health care needs for the 78 million Americans with limited income and resources [1]. Medicaid healthcare programs are usually joint funded through federal and state budgets and constitute a substantial proportion of the total federal and state budget. In 2018, total Medicaid spent $616 billion (9% of the total federal budget), of which $96 billion and $34 billion were incurred for children and maternal healthcare [2]. State-run Medicaid insurance programs finance nearly 50% of all U.S. births and postnatal care [3]. Although Medicaid comprises a substantial proportion of healthcare expenditures and is expected to increase by almost 6.2% per year, the overall health and wellbeing of Medicaid beneficiaries have not improved significantly [2,4]. Medicaid beneficiaries have higher rates of hospital usage (e.g., higher ED visits and readmission rates), higher mortality, and longer hospital stays than commercial insurance counterparts. Furthermore, Medicaid beneficiaries experience substantial racial and ethnic disparities in access to care (timely primary/specialist/OB-GYN care), preventive care (e.g., prenatal care visits and care experiences), and care experience [5,6,7,8,9]. Therefore, due to the pressure from increasing healthcare expenditure on the state budget and concern about care quality, many states have shown significant interest in managed care programs for the Medicaid population during the last decades [10]. Medicaid managed care organizations (MCOs) usually receive capacitated payments and are responsible for providing health benefits to their beneficiaries [11,12]. Since MCOs share most of the financial risk through a state-MCO contract structure, managed care programs are highly incentivized to reduce unwarranted costs, such as preventable hospital visits and medically unnecessary cesareans, while improving the quality of care. The total percentage of MCO enrollees has increased from 15 percent in 1995 to 79% in 2018 across 40 states [9,12,13,14].

The state of Florida has almost 30 years of experience with managed care in managing health benefits across its Medicaid population. In 1990, Florida started its first pilot risk-based managed care program in 2006 for 2 counties [15,16]. These programs led to significantly lower per capita healthcare expenditures than the other Medicaid programs [15,16]. These promising results in two counties boosted overall interest to enroll more Medicaid populations through managed care programs across all Florida counties. Consequently, Florida State Legislature passed mandated managed care legislation in 2011 and received federal waiver approval in 2013. Finally, in April of 2014, Florida state started to implement mandatory managed care for Medicaid beneficiaries through the Statewide Medicaid Managed Care (SMMC) program [10]. Most eligible Medicaid beneficiaries, including low-income families and their children, dual Medicaid-Medicare eligible enrollees, and people with certain disabilities, are required to participate in managed care programs to receive full benefits [10]. The exempt groups from managed care enrollment are women with certain primary care services (e.g., family planning and cancer screening), emergency Medicaid for non-American citizens, and children’s care in pediatric extended care centers [10]. After the implementation of the SMMC program, the managed care penetration rate experienced stable growth from 47% in September 2014 to 81.8% in December 2018 [17,18].

Due to the capitulated and performance-based state contract, Medicaid managed care programs are incentivized to provide beneficiaries with improved care access and initiate intervention programs addressing health determinants. Therefore, these initiatives could reduce costly unwarranted adverse health events and control the overall healthcare plan expenditure. Prior studies have reported improved access to preventive and primary care and a reduction in services, such as preventable ED visits and inpatient hospitalization, after the implementation of mandatory Medicaid managed care [19,20,21,22,23,24]. Studies have also reported a large reduction in preventable hospitalization and inpatient care related to ambulatory care-sensitive conditions [10,20,21]. In addition, Medicaid managed care was also found to be effective in reducing ED utilization, particularly in reducing racial/ethnic disparity, in preventable adult ED visit rates in Florida state [23,24,25,26]. However, most of these studies are limited to an estimation of the effect on preventable hospital visits. Limited information about the overall impact of managed care on maternal care exists, particularly regarding whether managed care programs impact obstetric hospital outcomes (e.g., primary cesarean and preterm birth) and associated racial/ethnic disparities in obstetric outcomes. Hence, we investigated the impact of the implementation of mandatory managed care with Medicaid on obstetric care outcomes. We sought to answer the following questions: first, what is the impact of Florida’s SMMC implementation on reducing obstetric hospital outcomes care? Second, is the SMMC program impacting the racial /ethnic disparities in obstetric hospital outcomes in Florida?

## 2. Materials and Methods

### 2.1. Data Source

The primary data source was the Hospital Cost and Utilization Project (HCUP) all-payer State of Florida ED visit and State of Florida Inpatient Databases (SIDs), from 1 January 2010 to 31 December 2017. The SED and SID database is an administrative all-payer database including the uninsured database, which is maintained and certified by the Agency for Health Care Research and Quality (AHRQ) [25]. The dataset contains patient-level information on demographic characteristics, insurance status, and International Classification of Diseases, Clinical Modification (ICD-9-CM and ICD-10-CM) diagnosis, and procedure codes from 265 acute care hospitals across 67 Florida counties. Medicaid quarterly market penetration data for Florida counties were obtained from the Medicaid monthly enrollment report published by the Florida Agency for Health Care Administration (AHCA) [17]. We calculated the quarterly rate using data from the monthly enrollment data in March, June, September, and December for the respective year [10,26]. The maternal delivery-related cohort for obstetric delivery-related admissions was identified from the diagnosis and procedure codes using the widely recognized stepwise methodology [26]. Patient discharges against medical advice, residential address outside the state, and in-hospital mortality were excluded from the analysis.

### 2.2. Outcome Variables

Our primary outcomes for maternal care were primary cesarean rates, preterm birth, postpartum preventable ED revisits, postpartum readmissions, and vaginal delivery after cesarean (VBAC) rates. These maternal care health outcomes are used to monitor the quality of obstetric care and vary disproportionally for historically disadvantaged race/ethnicities [27,28,29]. Furthermore, the reduction of primary/low-risk cesarean rates and preterm birth are major objectives set by the Healthy people 2030 goal. Primary cesarean rates were calculated as the percentage of livebirth cesarean deliveries among all obstetric low-risk deliveries. Primary cesarean deliveries were identified for all terms as singleton, vertex, and live birth deliveries without a prior cesarean [30]. Preterm birth was identified as the birth of an infant before 37 weeks of pregnancy using the ICD-9-CM and ICD-10 diagnosis codes. Postpartum hospital readmission was defined as an admission within 42 days (6 weeks) after the date of delivery admission [31]. Unplanned ED visits were calculated as a binary (yes/no) variable for any return to ED within 42 days of hospital discharge using the NYU-ED Billing Algorithm.

### 2.3. Statistical Analyses

Baseline categorical variables between pre-SMMC and pos-SMMC periods were compared using a Chi-square test and significance was interpreted as a *p* value less than 0.05. We used the difference-in-differences (DID) approach to evaluate the impact of SMMC on pediatric and maternal healthcare quality outcomes. The DID approach is a quasi-experimental design that has been widely used in causal relationships in health care policy research, particularly when randomization is not available [32,33,34,35]. We compared the changes in selected maternal health outcomes among Medicaid beneficiaries (treatment) and the commercially insured population (comparison), before and after the implementation of SMMC. The estimation in our analysis assumed that the trend in health outcomes for commercially insured patients reflects the secular trend in outcomes [10,26]. We considered the pre-SMMC period (January 2010 to March 2014) and comparator post-SMMC implementation period from October 2014 to September 2017, excluding the implementation periods of April 2014 to September 2014. Racial-ethnic disparities in Medicaid enrollees (African American, non-Hispanic, and Hispanic patient groups) are defined as the health outcome difference when compared with the outcomes of commercially insured white non-Hispanic patients. We performed multivariable linear regression analyses with county and quarter as fixed effects and robust SEs to determine health outcome differences between the before and after SMMC implementation periods. The formal DID estimation model for other health outcomes is as follows:

$$Y_{ijkl}=\beta_{o}+\beta_{1}Medicaid_{ijkl}+\beta_{2}Medicaid_{ijkl}\times Post{\mathrm{SMMC}}_{ijkl}\;+\gamma_{k}Medicaid_{ijkl}\times Race/ethnicity_{ijkl}\times Post{\mathrm{SMMC}}_{ijkl}+qtr_{l}+county_{i}+\delta X_{ijkl}+\varepsilon_{ijkl}$$

where $Y_{ijkl}$ is the binary (yes/no) indicator for the selected outcome for patient *i*, county *j*, race/ethnic group *k*, and quarter *l.* $Post{\mathrm{SMMC}}_{i}$ and $Medicaid_{i}$ are the binary (yes/no) indicators for the race/ethnicity, post-SMMC implementation period, and Medicaid enrollee, respectively. Patients’ race/ ethnicity was grouped into white non-Hispanic (reference group), African American non-Hispanic, and Hispanic. $county_{i}$ and $qtr_{l}$ represent the fixed effect that quarter and county might have on the outcome and therefore, reduce the estimation bias. $X_{i}$ represents covariate variables for patient i and the covariates for maternal care outcomes are age group (<18, 18–30, 30–40, >40 years), weekdays/weekends, and obstetric comorbidity scores. We evaluated 24 common comorbidities and weighted summed them to categorize them into 3 patient groups (0 (lowest risk), 1 or 2, or >2 (highest)) [36].

The key variables of interest were $\beta_{2}Medicaid_{ijkl}\times Post{\mathrm{SMMC}}_{ijkl}$ and $\gamma_{k}Medicaid_{ijkl}\times Race/ethnicity_{ijkl}\times Post{\mathrm{SMMC}}_{ijkl}$. If the SMMC program improved maternal health outcomes compared with the pre-SMMC period (relative to the privately insured patient), then we would observe a negative regression coefficient in $Medicaid_{ijkl}\times Post{\mathrm{SMMC}}_{ijkl}$ terms. Similarly, if there was an improvement in health outcomes for racial/ethnic minority Medicaid enrollees relative to whites in Florida, we would observe negative statistically significant coefficients for the $Medicaid_{ijkl}\times Race/ethnicity_{ijkl}\times Post{\mathrm{SMMC}}_{ijkl}$ terms. All statistical analyses were performed using R studio, and a 2-sided *p*-value less than 0.05 was considered statistically significant.

## 3. Results

The analysis included 1,749,129 hospital births by Florida residents from 1 January 2010 to 31 December 2017. Among these hospital births and those that met our exclusion criteria, our analysis identified 1,499,994 index births with a median age of 28 years. Of these index hospital births, 584,604 (38.9%) and 122,566 (8.1%) were cesarean deliveries and preterm births, respectively. The VBAC birth rate was 5.6% among Florida residents with a prior cesarean birth. The postpartum readmission and ED visit rates for the readmissions were 2.0% and 3.5%, respectively. The majority (51.9%) of the births were covered by Medicaid. The total hospital charges for cesarean deliveries in Florida during 2010–2017 were $12.9 billion, with average annual hospital charges of $1.61 billion. Particularly, the annual total hospital charges for Medicaid-insured cesarean delivery were $819 million for the study period. In addition, the total postpartum readmission and unplanned ED visit charges were $550 million and $41 million, respectively.

Table 1 shows the summary statistics of the patient characteristics of the group overall, in the pre-period and post-period of the implementation of SMMC. Patients in the pre-SMMC groups tended to be younger (mean age: 27.8 vs. 28.5, *p* < 0.05) and more likely to be covered by Medicaid (53.3% vs. 50.3%, *p* < 0.05), compared to the privately insured. Patients in the pre-SMMC groups were also more likely to have lower comorbidity scores (82.0%, 77.0% for comorbidity score 0). Patients in the pre-SMMC period also tended to be more likely (3.7% vs. 3.4%, *p* < 0.05) to visit ED after birth compared with the post-SMMC period.

**Figure 1:**
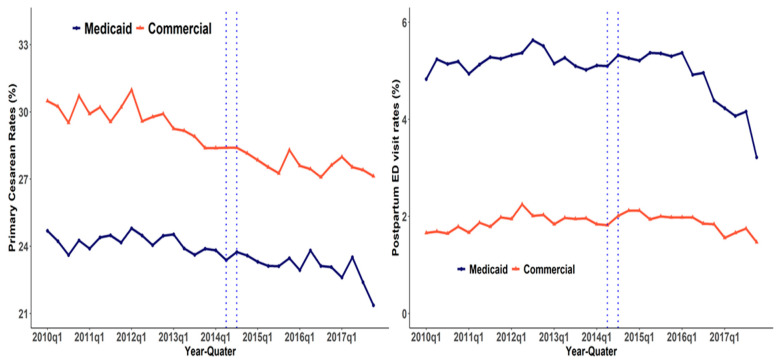
Trends in the primary cesarean rates and postpartum ED visits by payor.

**Figure 2:**
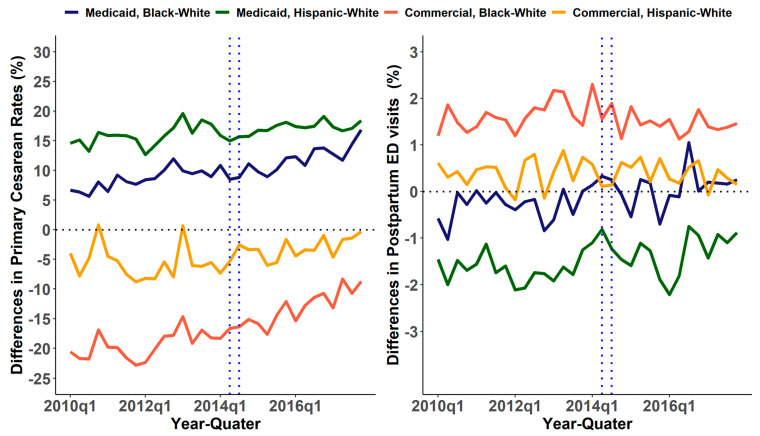
Disparities in primary cesarean rates and postpartum ED visits between non-Hispanic white and non-Hispanic African American and non-Hispanic white and Hispanic patients with Medicaid or commercial insurance.

Figure 1 shows the trends in the primary cesarean rates and postpartum ED visit rates by payor. Primary cesarean rates remained almost the same from 2010 until Q4 of 2013 and started to decrease from Q1 of 2014 for Medicaid and commercially insured mothers. The gradient of the decrease among Medicaid cover births was slightly higher than the commercial counterparts. Postpartum ED visit rates increased from 2010 until Q2 of 2015 and a downward trend was observed after Q3 of 2015. Figure 2 shows the trends in the disparities in primary cesarean rates and postpartum ED visit rates by payor. The disparities in primary cesarean rates increased from 2010 until Q1 of 2014 and a slight upward trend in the disparities after Q1 of 2015 was shown. Similarly, an increasing upward trend was observed for Hispanic Medicaid births compared with white commercial births. The pattern of the other disparities of postpartum preventable ED visits was less clear.

The result from the difference-in-difference method used to compare obstetric outcomes is shown in Table 2. After adjusting for patient characteristics, the results of the DID specification indicated that Medicaid-enrolled mothers experienced a significant 9% and 6% lower incidence of primary cesarean (incidence rate ratio (IRR) = 0.91, 95% confidence interval (CI) 0.88–0.93) and preterm births (IRR = 0.94, 95% CI 0.91–0.98), respectively, compared with their commercially insured counterparts. Furthermore, Medicaid-enrolled mothers experienced a 14% and 13% lower incidence of being readmitted (IRR = 0.86, 95% CI 0.80–0.93) and revisiting ED (IRR = 0.87, 95% CI 0.82–0.93) after SMMC implementation compared with the pre SMMC period. Overall, Medicaid VBAC rates remained unchanged and were not found to be significant between the two comparator periods. However, implementation of SMMC was not found to be significantly associated with a reduction in the selected obstetric outcomes for racial/ethnic minorities relative to whites. In the DID estimation, primary cesarean rates for Medicaid Hispanic (IRR = 1.14, 95% CI 1.08–1.19) patients were increased relative to that of non-Hispanic white Medicaid patients after the implementation of the SMMC program. A similar significant increase in postpartum ED visit rates was observed for non-Hispanic black (IRR = 1.13, 95% CI 1.02–1.26) mothers after delivery hospitalizations. Racial/ethnic disparities in all other obstetric outcomes were not significantly associated with an increase or decrease after the SMMC implementation. We found similar significant reductions in racial/ethnic disparities for potentially preventable ED visits (non-Hispanic African American, IRR = 0.83, 95% CI 0.72–0.96; Hispanic IRR = 0.73, 95% CI 0.60–0.88).

## 4. Discussion

In summary, we investigated the impact of mandatory managed care programs in the state of Florida on the reduction of several obstetric hospital outcomes and associated persistent racial/ethnic disparities. To our knowledge, this is the first study to analyze SMMC’s impact on obstetric care quality and explore the association between SMMC implementation and racial/ethnic disparities. Our DID estimation showed evidence of a substantial reduction in several obstetric care outcomes for the Medicaid population compared with the privately insured patient population. However, our results did not provide any evidence of a reduction in disparities in obstetric care outcomes, including primary cesarean rates and preterm births, compared with white and privately insured children. In general, this study highlights the overall impact of SMMC on obstetric healthcare quality in Florida and provides important insights regarding the positive dynamics and potential scope for improvement in care quality and associated racial/ethnic disparities in the Medicaid population.

In our study, we found a significant reduction in primary cesarean rates and preterm births after the implementation of SMMC programs. This overall reduction in primary cesarean and preterm births reported in our study can be explained by multifactorial causes: (1) many managed care organizations implemented effective intervention strategies, such as need-based assistance programs [12,13,14]; and (2) improved access to more primary care physicians and pediatricians within the MCO network for the vulnerable population, due to higher market competition [15,16]. The reduction in postpartum hospital utilization after SMMC implementation is consistent with the obstetric outcomes reported in prior managed care cohorts [37,38,39]. The decline in postpartum readmission rates after SMMC suggests an improvement in postnatal primary care access, hospital discharge, and care continuity after birth [40,41,42]. Although SMMC resulted in an overall reduction in postpartum preventable hospital visits, the slight increase in disparities in non-Hispanic black patients compared with white counterparts reinforces the need for more targeted interventions addressing the persistence of racial/ethnic disparities in obstetric care and birth outcomes. The significant increase in primacy cesarean disparities in the Hispanic patient population is likely related to a persistence in cultural differences, patient preferences, and attitudes towards the mode of delivery [43,44]. In addition, the language barrier may contribute to poorer healthcare access and adverse health outcomes for Hispanic/ Latino mothers, particularly those living in households with limited English proficiency [45,46,47,48]. Additionally, the prevalence of higher cesarean-inclined demographic subgroups (e.g., Latin Americans and West Indian Americans in Florida) may contribute to the similar increased cesarean rate among Hispanic mothers [49,50]. Therefore, implementing high-risk population-specific nonclinical interventions, such as midwife/doula-led continuity of care, antenatal education, and training for patients with low-risk pregnancies, through Medicaid programs can significantly reduce the primary cesarean rate and other potential adverse birth outcomes [51,52].

Evidence of a substantial reduction in several maternal care outcomes in the Medicaid population indicates the promising success regarding maternal care quality under the SMMC program. These findings are particularly important since SMMC MCOs reported a substantial financial loss of $550 million throughout the 5-year contracts [53]. Hence, it is critical for all stakeholders, including state government, MCOs, and relevant stakeholders, to understand the impact of SMMC on the quality of care and understand the opportunities for improvements. Consequently, our study offers critical insights into the pediatric and maternal care quality after the implementation of SMMC in Florida and, therefore, suggests opportunities for both MCOs and policymakers to improve the overall quality of care and reduce avoidable healthcare expenditures. The similar rates of preventable readmission in both pediatric and maternal care suggest unresolved systemic care transition complications resulting from fragmented care [54,55]. Through an understanding of the SMMC impact pattern, MCOs could implement strategies to engage certain patients through alternative care settings, such as Telehealth and home health care initiatives [56,57]. Florida MCOs have broad flexibility to cover services and set their requirements and, therefore, the findings of our study could help MCOs to design appropriate alternative care settings to support vulnerable populations by providing the care they need [58,59].

This study has several common limitations, most of which are related to the retrospective analysis of administrative claim databases. First, the HCUP database is an administrative claim dataset that uses ICD codes to classify patients’ medical diagnoses, procedures, and outcomes. The possibility of coding inaccuracy or incorrect information cannot be dismissed. Second, although our study makes a significant contribution by investigating the impact of mandatory managed care in Florida state MCO settings, the findings of this study may not be generalizable to other U.S. state MCOs. Third, the HCUP dataset does not include information about federal hospital discharges and non-hospital births (e.g., birth center deliveries and home births). However, the proportion of these out-of-hospital births is small compared to hospital births and would not affect our estimation [60]. Fourth, we only considered ED revisits for different insurance payers after the ED and inpatient care were covered by Medicaid managed care. However, the proportion of heterogeneous insured ED revisits was expected to be low compared with the same insurance ED revisits. Finally, the HCUP dataset does not include patient-reported personal characteristics, socioeconomic information, or disability status. The inclusion of these variables with propensity score adjustment in the analysis might have improved the estimation accuracy. Future research should be directed towards SMMC’s impact on pregnancy well-care visits to better understand the holistic impact of mandatory managed care in Florida.

## 5. Conclusions

Due to the capitulated and performance-based state contract, Medicaid managed care programs are incentivized to provide beneficiaries with improved maternal access to care and address health determinants, and therefore reduce obstetric/postpartum health outcomes and associated disparities. Using a robust health policy impact analysis, we analyzed and quantified the impact of the implementation of the Statewide Medicaid Mandatory Managed Care (SMMC) programs on reducing obstetric hospital and postpartum health outcomes, particularly focusing on persistent racial/ethnic disparities. Our study provided evidence of a substantial reduction in several maternal care outcomes in the Medicaid population, indicating the promising success regarding maternal care quality under the SMMC program. In addition, our findings also suggest that the implementation of SMMC did not result in any significant reduction in racial/ethnic disparities in obstetric outcomes. As such, our study highlights the comprehensive impact of SMMC implementation on obstetric outcomes and provides important insights to stakeholders about existing realities and potential scope for improvement in obstetric care quality and associated health disparities.

## Figures and Tables

**Table 1:** Baseline characteristics of the patients overall, in the pre-period and post-period of the implementation of mandatory managed care.

	Overall, N = 1,499,994, n (%)	Pre SMMC Period N = 791,718, n (%)	Post SMMC Period N = 662,981, n (%)	*p* Value
Cesarean delivery				
Yes	584,604(38.9)	310,779 (39.3)	256,113 (38.6)	*p* < 0.05
No	915,390 (61.1)	480,939 (60.7)	4,066,868 (61.4)	
Primary cesarean delivery				
Yes	313,127 (20.8)	170,287 (21.5)	133,524 (20.1)	*p* < 0.05
No	118,686 (79.2)	621,431 (78.5)	529,457 (79.9)	
Preterm birth				
Yes	122,566 (8.1)	57,266 (7.2)	62,488 (9.4)	*p* < 0.05
No	1,377,428 (91.9)	734,452 (92.7)	600,493 (90.6)	
Postpartum readmission				
Yes	29,872 (2.0)	14,882 (1.9)	141,21 (2.1)	*p* < 0.05
No	1,470,122(98.0)	776,836 (98.1)	648,860 (97.9)	
Postpartum ED visit				
Yes	53,152 (3.5)	29,183 (3.7)	22,351 (3.4)	*p* < 0.05
No	1,446,842 (96.5)	762,535 (96.3)	640,630 (96.6)	
Prior Cesarean				
Yes	287,554 (19.2)	147,551 (18.6)	131,124 (19.7)	*p* < 0.05
No	1,212,440 (80.8)	644,167 (81.4)	531,857 (80.3)	
Vaginal delivery after cesarean				
Yes	16,077 (1.1)	7059 (0.9)	8535 (1.3)	*p* < 0.05
No	1,483,917 (98.9)	784,659 (91.1)	654,446 (98.7)	
Age (years)				
<18	27,026 (1.8)	17,608 (2.2)	8743 (1.3)	*p* < 0.05
18–30	858,875 (57.2)	465,166 (58.8)	368,082 (55.5)	
30–40	566,371 (37.8)	284,143 (35.9)	264,686 (39.9)	
>40	477,22 (3.2)	24,801 (3.1)	21,470 (3.2)	
Race/ethnicity				
Non-Hispanic white	724,174 (48.3)	385,544 (48.7)	316,442 (47.7)	*p* < 0.05
Non-Hispanic Black	347,720 (23.2)	186,190 (23.5)	151,323 (22.8)	
Hispanic	329,438 (22.0)	168,357 (21.3)	151,295 (22.8)	
Others	79,979 (5.3)	41,697 (5.3)	35,721 (5.3)	
Payor				
Medicaid	778,662 (51.9)	421,623 (53.3)	333,341 (50.3)	*p* < 0.05
Commercial	646,442 (43.1)	326,714 (41.3)	300,091 (40.3)	
Others	74,890 (5.0)	43,381 (5.4)	29,549 (4.4)	
Comorbidity score				
0	1,196,338(79.8)	649,319 (82.0)	510,534 (77.0)	*p* < 0.05
1–2	232,181 (15.5)	112,014 (14.1)	113,321 (17.1)	
>2	71,475 (4.8)	30,385 (3.8)	39,126 (5.9)	
SMMC: Statewide Mandatory Managed Care				

**Table 2:** Difference-and-difference (DID) effect estimations on obstetric hospital outcomes.

Outcomes	Overall Insurance × PostSMMC	Racial/Ethnic Disparities Insurance × Race/Ethnicity × PostSMMC	
	Medicaid Beneficiaries Incidence Ratio (95% CI)	Medicaid Non-Hispanic Black Incidence Ratio (95% CI)	Medicaid Hispanic Beneficiaries Incidence Ratio (95% CI)
Primary cesarean rates	**0.91 (0.88–0.93)**	1.04 (0.99–1.10)	**1.14 (1.08–1.19)**
Preterm birth rates	**0.94 (0.91–0.98)**	1.03 (0.97–1.05)	1.05 (0.97–0.98)
Postpartum ED visits	**0.87 (0.82–0.93)**	**1.13 (1.02–1.26)**	1.07 (0.96–1.21)
Postpartum preventable readmissions rates	**0.86 (0.80–0.93)**	1.11 (0.98–1.26)	1.02 (0.88–1.17)
Vaginal birth after cesarean (VBAC) rates	1.02 (0.92–1.12)	1.04 (0.86–1.26)	1.11 (0.90–1.36)

Notes: This table reports regression results from difference-in-difference estimation using generalized linear estimation. Control variables for the equation for primary cesarean, preterm birth, postpartum ED visits, postpartum preventable readmissions, and vaginal birth after cesarean outcomes included patients’ age, insurance (commercial (reference group), Medicaid), race/ethnicity (white non-Hispanic (reference group), black non-Hispanic, Hispanic), comorbidity score (0 (reference group), 1–2, nights, and >2), county fixed effect, and quarter fixed effects. Moreover, 95% confidence intervals in brackets were adjusted for clustering within counties of patients’ residence. Statistically significant (*p* < 0.05) incidence ratio estimates are highlighted in bold.

## Data Availability

In this research, limited datasets were used, and datasets are available through the Agency for Health Care Administration, Florida. Website: https://www.floridahealthfinder.gov/researchers/orderdata/order-data.aspx (accessed on 3 April 2021).
